# Application of Nanosize Zeolite Molecular Sieves for Medical Oxygen Concentration

**DOI:** 10.3390/nano7080195

**Published:** 2017-07-25

**Authors:** Mingfei Pan, Hecham M. Omar, Sohrab Rohani

**Affiliations:** Department of Chemical and Biochemical Engineering, Faculty of Engineering, University of Western Ontario, London, ON N6A 5B9, Canada; pmf19516@gmail.com (M.P.); homar7@uwo.ca (H.M.O.)

**Keywords:** nanosize zeolite, microporous adsorption, bidisperison dynamic model, portable oxygen concentrator, COMSOL Multiphysics

## Abstract

The development of a portable oxygen concentrator is of prime significance for patients with respiratory problems. This paper presents a portable concentrator prototype design using the pressure/vacuum swing adsorption (PVSA) cycle with a deep evacuation step (−0.82 barg) instead of desorption with purge flow to simplify the oxygen production process. The output of the oxygen concentrator is a ~90 vol % enriched oxygen stream in a continuous adsorption and desorption cycle (cycle time ~90 s). The size of the adsorption column is 3 cm in diameter and 20 cm in length. A Li^+^ exchanged 13X nanosize zeolite is used as the adsorbent to selectively adsorb nitrogen from air. A dynamic model of the pressure and vacuum swing adsorption units was developed to study the pressurization and depressurization process inside the microporous area of nanosized zeolites. The describing equations were solved using COMSOL Multiphysics Chemical Engineering module. The output flow rate and oxygen concentration results from the simulation model were compared with the experimental data. Velocity and concentration profiles were obtained to study the adsorption process and optimize the operational parameters.

## 1. Background

Air pollution is becoming a worldwide problem particularly in heavily populated cities like Beijing, Los Angeles, and Mexico City. Air pollution leads to acute and chronic respiratory problems that are becoming a growing concern from both global and individual levels. Wearing disposable face masks has become a popular method to get some protection from exposure to pollutants. However, for people with poor autonomous respiration ability, like chronic obstructive pulmonary disease (COPD) patients, it is hard to breathe through disposable face masks. As a result, there is a growing need to address the health and quality of life through a light-weight and portable oxygen concentrator with a medical grade oxygen supply (O_2_ concentration: ~88–92 vol %). Currently, oxygen concentration techniques are based on air separation processes such as cryogenic technique, membrane separation and pressure swing adsorption (PSA) that can effectively produce enriched oxygen using ambient air as the source. Although cryogenic distillation acts as the leading process for air separation in an industrial oxygen plant, the pressure swing adsorption (PSA) technique has the advantage of reducing energy consumption for small production scales. Compared to the membrane separation technique, which requires higher pressure and expensive selective permeable materials, pressure swing adsorption using nanosize zeolite adsorbents is a more feasible solution for portable oxygen concentrator development. Due to the large microporous surface area inside the zeolite adsorbents, the portable oxygen concentrator using the PSA technique can adsorb nitrogen from air and output an enriched oxygen stream under high-pressure conditions. The concentrator can be regenerated by decreasing the pressure to release the adsorbed nitrogen. In this way, a continuous oxygen stream supply can be generated to assist an individual and increase the fraction of inspired oxygen. The oxygen concentration system is usually coupled with a filtration system to remove PM 2.5 and improve the quality of product gas stream.

The pressure swing adsorption process was patented by Finlayson and Sharp in 1932 (GB Patent 365092) describing an air separation process with pressurization adsorption and depressurization desorption steps [1]. In 1958, two different pressure swing adsorption units were patented: one using fractional product purge flow for desorption step (Skarstrom, 1958) and the other using pressure vacuum adsorption (PVSA) step (Montgareuil and Domine, 1958) [2,3].

The selectivity for zeolite adsorbents to adsorb nitrogen compared to oxygen is due to the interaction between electrostatic field of the cationic zeolite and the quadrupole moment of the nitrogen and oxygen. The quadrupole of nitrogen is three times higher than that of oxygen, which leads to a selective adsorption onto the zeolite surface [4]. The most common type of commercial zeolite for oxygen concentration process is zeolite 13X due to its outstanding nitrogen to oxygen adsorption selectivity. However, 13X zeolite modified with Li^+^ exchange method exhibits a higher nitrogen adsorption capacity at the active cation sites of the zeolite framework [5,6,7]. JLOX-101 LiX zeolite provided by LUOYANG JIANLONG Chemical is designed specifically for a PVSA process with high oxygen selectivity [8].

The PSA system was first installed in industrial plants as a replacement for cryogenic separation plants due to lower capital and operating costs. Portable pressure swing adsorption oxygen concentrators have been designed and developed in the past two decades due to the synthesis of more effective zeolites with higher gas adsorption capacity and selectivity. The PSA oxygen concentrator is able to provide a continuous oxygen flow using the ambient air as the gas source. Two-bed or multi-bed PSA systems are utilized alternating adsorption and desorption operations of the zeolite columns to achieve continuous oxygen separation. For on-site medical grade oxygen production, a cylinder gas source or several powerful air compressors are usually installed for the rapid pressure swing adsorption process to maintain the productivity of high purity oxygen stream [9,10,11]. Considering the mobility of small-scale concentrators, a variety of simplified designs would be added to the pressure swing adsorption process for system miniaturization.

In this paper, we compare the performance of a portable miniature PVSA oxygen concentrator unit with two adsorption columns, an air compressor, four solenoid valves and a back-pressure regulator (total weight less than 2 kg) built in our laboratory with the simulation results under modified Montgareuil and Domine cycle using COMSOL Multiphysics Chemical Engineering module. Compared to the conventional PSA cycle using purge flow with intermediate evacuation pressure (−0.6 barg) to regenerate the adsorption column, the deep vacuum (−0.82 barg) desorption step of the PVSA cycle reduces the column and compressor size from the perspective of industrial prototype design. A dual-head air compressor is selected to generate pressure and vacuum condition simultaneously for the dual-column adsorption unit, which simplifies the gas source generation. The oxygen concentrator unit can provide a ~90 vol % enriched oxygen stream to meet the concentration requirement of medical-grade oxygen.

## 2. Materials and Methods

**Figure 1:**
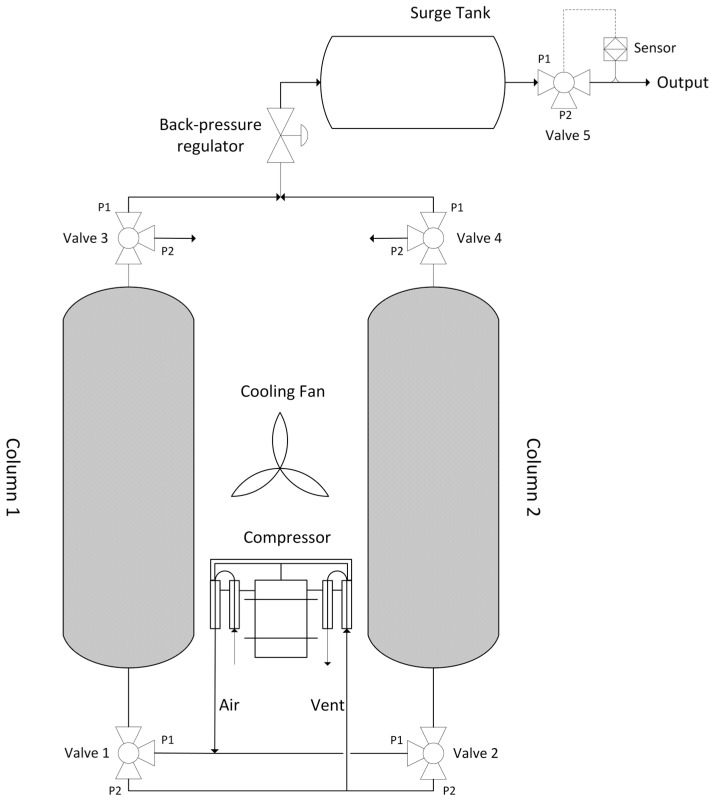
Schematics of the PSA oxygen concentrator.

**Figure 2:**
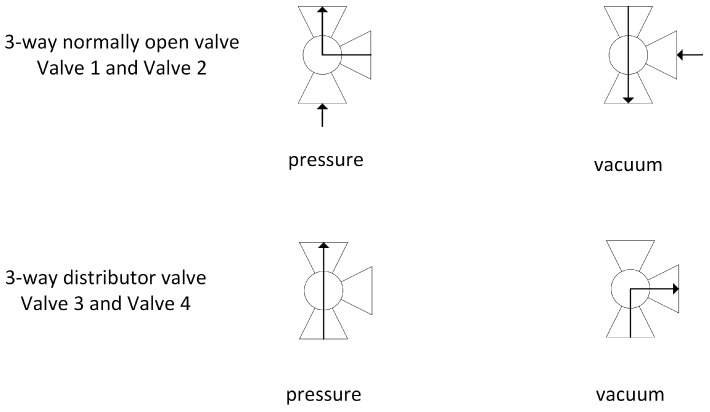
Flow directions Schematics of the normally open and distributor three-way valve under pressure and vacuum conditions.

**Figure 3:**
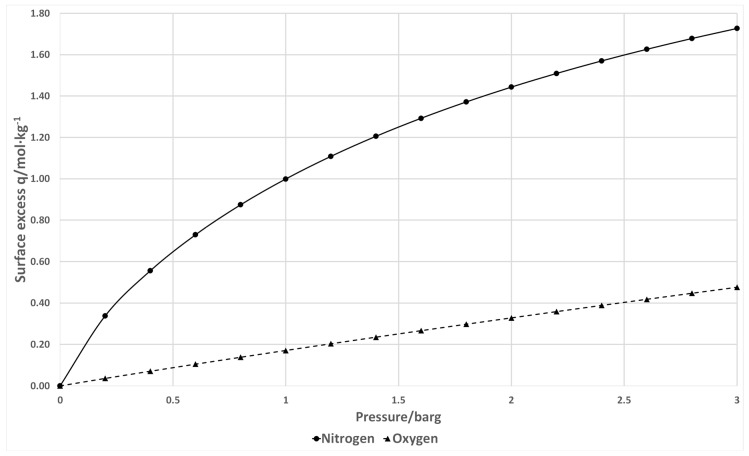
Adsorption equilibrium isotherms for nitrogen and oxygen onto zeolite LiX at 20 °C. Reproduced with permission from [17]. Industrial & engineering chemistry research, 2008.

Figure 1 shows the schematic of the oxygen concentrator using a two-column PVSA cycle. The concentrator consists of two adsorption zeolite columns, a dual-head air compressor (Parker Hannifin Corp., TTC IIS Miniature Diaphragm Pump, Hollis, NH, USA), five three-way solenoid valves (Parker Hannifin Corp., X-Valve, Hollis, NH, USA), a back-pressure regulator, an oxygen sensor (Teledyne GB300 Oxygen analyzer, Thousand Oaks, CA, USA), a surge tank, a pressure sensor (Freescale Semiconductor, MPXV7002DPT1CTND, Austin, TX, USA) and a cooling fan (avoiding the temperature rise of the air compressor) [12,13,14,15]. The oxygen sensor is an electrochemical transducer named Micro-fuel Cell calibrated by the Faraday type calibration method based on the product gas from electrolysis of water [16]. The valve sequence is shown in Table 1. The concentrator operation consists of four different steps. In step 1, column 1 is pressurized by the compressor while column 2 is connected to ambient air to decrease the pressure. In step 2, the pressure inside column 1 reaches the working pressure (1.79 barg) with enriched oxygen flowing out through the backpressure regulator (set at 1.79 barg) to the surge tank. Meanwhile the pressure in column 2 decreases to vacuum (set at −0.82 barg) via the air compressor to regenerate the zeolite. In steps 3 and 4, the adsorption and desorption positions of column 1 and 2 are reversed to regenerate the saturated column 1 and pressurize the regenerated column 2. By switching the valve sequence (step 1 → 4 → 1), a continuous enriched oxygen stream can be obtained from the surge tank. The flow directions of the two different types of valves (normally open and distributor three-way solenoid valves) are shown in Figure 2 with two different roles in the pressure swing operations. Valve 1 and 2 are normally open 3-way valves directing the pressurizing flow and countercurrent blowdown flow while valve 3 and 4 are distributor 3-way valves for pressurizing and depressurizing port control at the column end. According to the adsorption isotherm of zeolite LiX shown in Figure 3 (Santos et al., 2008), zeolite capacity and selectivity of nitrogen to oxygen is higher under pressure vacuum swing range compared to conventional pressure swing [17]. In order to reduce the compressor size, a dual-head air compressor is considered to perform pressurization and vacuum operation simultaneously. By sharing the central motor, two diaphragms can separately provide pressurized gas and evacuation pressure. Compared to the size and total power consumption of two diaphragm compressors, a dual-compressor is less bulky (25% size reduction) and more efficient (12% input power saving), which benefits the miniaturization of the prototype. Summarizing the system design, a PVSA process that uses a vacuum for desorption and pressurization for adsorption can achieve relatively high capacity, allowing more nitrogen adsorption while using a smaller compressor for gas supply.

**Figure 4:**
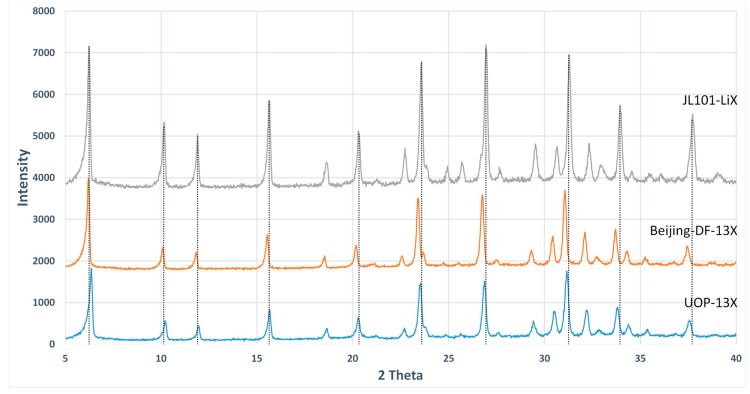
XRD pattern of three different types of zeolites: UOP-13X, Beijing-DF-13X and JLOX101-LiX.

**Figure 5:**
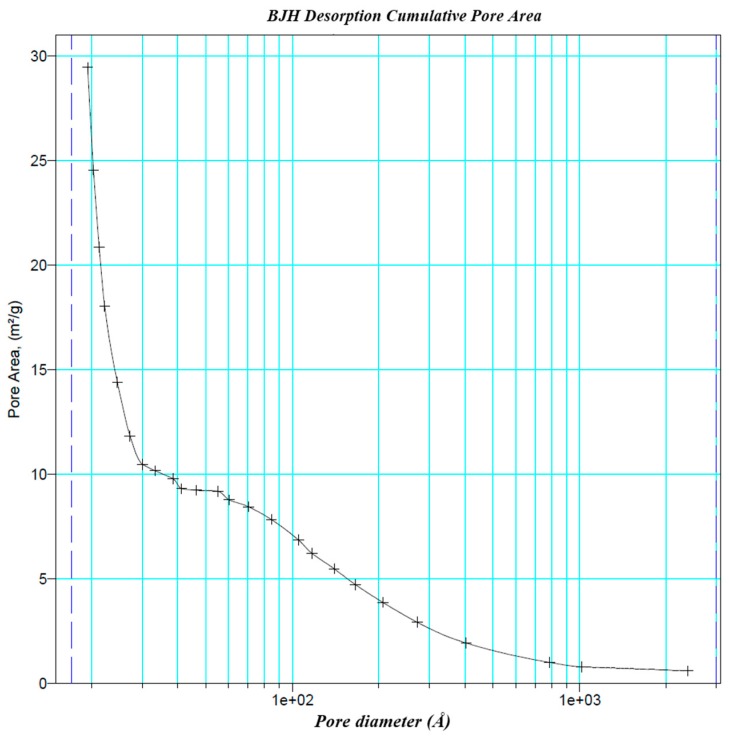
BJH adsorption cumulative pore area distribution of zeolite JLOX101-LiX.

**Figure 6:**
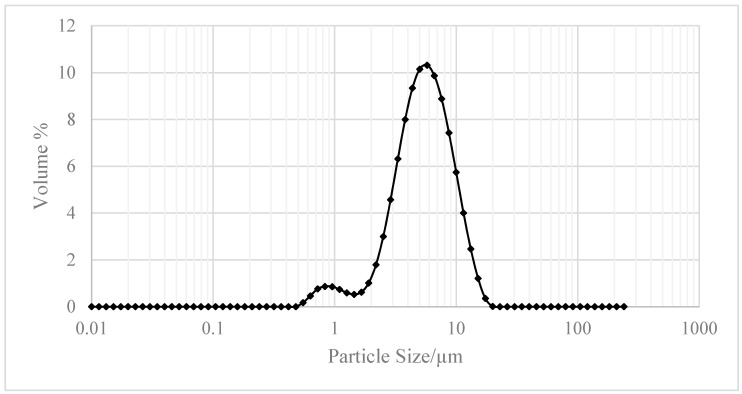
Particle size distribution of zeolite JLOX-101-LiX powder sample.

Zeolite JLOX-101 (Ø0.4–0.8 mm) from JIANLONG CHEMICAL (Yanshi, Henan, China) was used as the adsorbent inside the adsorption column based on the zeolite characterization [14]. Zeolite JLOX-101 is a Li^+^ exchanged zeolite 13X designed for industrial oxygen concentration. Figure 4 exhibits the powder XRD (PXRD) patterns of zeolite JLOX-101 with two types of sample zeolite 13X from UOP (Des Plaines, IL, USA) and Beijing-DF (Beijing, China) [18,19]. The PXRD spectra of the collected solid forms were obtained on a 2500 diffractometer (D/MAX, Rigaku, Japan) with a Cu Kα radiation source (λ = 1.5406 Å) at 100 mA and 40 kV. The samples were recorded at a scanning rate of 5° per minute over a 2θ range of 5–40°.The XRD peak position and shape of zeolite JLOX101-LiX are essentially the same as those of zeolite 13X, which means zeolite JLOX101-LiX maintains the intrinsic crystalline structure of zeolite 13X after the Li^+^ ion-exchange method. Table 2 shows the BET adsorption results of zeolite JLOX-101 with the sample 13X zeolite from Beijing-DF through ASAP 2010 (Micromeritics Instrument Corporation, Norcross, GA, USA). Both the BET surface area and micropore volume of JLOX-101 zeolite are larger than those of 13X zeolite (Table 2) and the adsorption average pore diameters of zeolite JLOX-101 and 13X-BJ-DF are 21.26 Å and 22.36 Å, respectively, which are in agreement with the microporous nitrogen adsorption theory of X type zeolite. Figure 5 illustrates the Barrett–Joiner–Halenda adsorption cumulative pore area using nitrogen as the analysis adsorptive. The nitrogen occupies most of the microporous surface area (pore diameter <2 nm) and small part of the mesoporous surface area (pore diameter 2~50 nm). Figure 6 depicts the particle size distribution of the zeolite powder samples through MASTERSIZER 2000 (Malvern Instruments Ltd, Worcestershire, UK). The diameter of the vol % peak (5.75 µm) is used as the average microparticle diameter in the bidispersion model for mathematical simulation.

Due to the negatively charged zeolite surface, the polar compounds H_2_O and CO_2_ in the air are preferentially adsorbed and occupy the effective zeolite micropores during the PSA process. Conventional vacuum desorption cannot fully desorb the H_2_O and CO_2_ molecules, which gradually decrease the zeolite capacity under the cyclic operation of adsorption and desorption steps. In order to improve the lifespan of the adsorption column, a thin layer of activated alumina was added before the zeolite layer as a desiccant to prevent zeolite deactivation. The volume ratio of the desiccant to whole column was 0.15–0.20 (Chai et al., 2011) using ambient air source (0.04 vol % CO_2_ and 0.4–0.8 vol % moisture) [20].

Table 3 shows the physical properties of the zeolite, desiccant and adsorption column. The bed voidage and particle voidage are estimated from the voidage variation study and single point BET adsorption total pore volume, respectively [21]. The adsorption constants for zeolite column are fitted with the experimental data based on the single particle adsorption isotherms of LiX zeolite. The PVSA system operates between 1.79 barg for adsorption and −0.82 barg for desorption under a compressor flow. The time for different steps of the column test is summarized to maintain a stable oxygen flow and achieve a complete regeneration of column in each cycle. The experimental performance of the column would be compared to the model prediction in the following paragraphs.

## 3. Mathematical Model

### 3.1. Transport and Adsorption Equations

In order to study the performance of the adsorption column, a three-dimensional model was developed using COMSOL Multiphysics. The equations were solved using the finite element method. Due to the cylindrical geometry of the adsorption column, a two-dimensional axi-symmetrical approximation was used to decrease the computational cost. The following assumptions were made in the model equations:

(a) The air source is assumed to be a binary ideal gas with 79% N_2_ and 21% O_2_.
(b) Uniform bed voidage and particle diameter.
(c) The stabilized gas temperature at inlet and outlet is measured to be 22.5 and 23.8 °C by thermocouple, checking the temperature dependent adsorption model (Santos et al., 2008), the nitrogen and oxygen capacity difference (error = $\frac{\left|q_{i,22.5{}^{\circ}C}-q_{i,23.8{}^{\circ}C}\right|}{q_{i,22.5{}^{\circ}C}}\times 100\%$) at 1.79 barg is calculated to be 1.47% and 1.03%, respectively, which is negligible compared to the adsorption equilibrium equations, so the system is assumed to be isothermal [17].
(d) Gravity effects on fluid flow are negligible.

**Figure 7:**
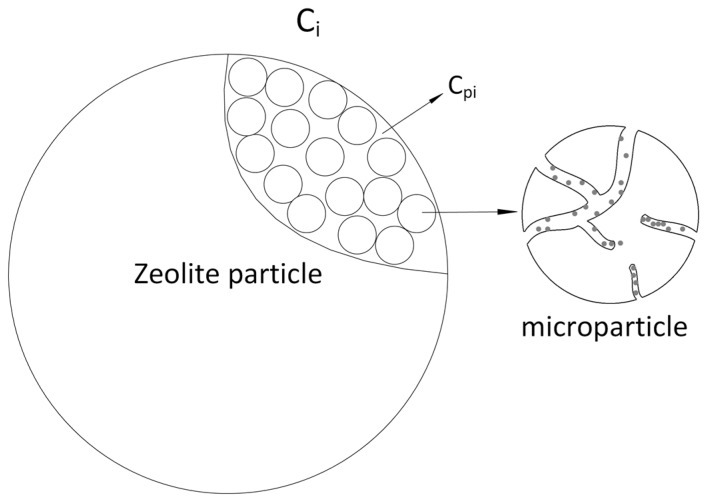
Schematics of the zeolite particle structure.

The commercial zeolite particles are produced by aggregation of nanosize particles (zeolite powder) using a binder. The structure of the zeolite particles is shown in Figure 7. In the pressure swing process, the high-pressure flow will first disperse from the column void to the macropore area inside the nanosize zeolite particles represented by the macropore dispersion equations. The gas adsorption usually happens at the micropore surface of the zeolite microparticles, which is simulated by the adsorption mass transfer equations in the model. Based on the characteristics of the commercial zeolites, the mass transfer of adsorption model is controlled by a set of bidispersion equations [26].

The velocity profile of the gas stream was developed in the Free and Porous Media Flow Interface of COMSOL Multiphysics using the Navier–Stokes Equation to describe the flow in open regions, and the Brinkman equation to describe the flow in porous media.

The flow in zeolite micropore area is governed by a set of the continuity equation and the momentum equation, which together form the Brinkman equations (COMSOL, 2016) [27]:

(1) $$\frac{\partial }{\partial t}(\varepsilon_{c}\rho )+\nabla (\rho u)=Q_{br}$$

(2) $$\frac{\rho }{\varepsilon_{c}}\left(\frac{\partial u}{\partial t}+(u\cdot \nabla )\frac{u}{\varepsilon_{c}}\right)=-\nabla p+\nabla \cdot \left\{\frac{1}{\varepsilon_{c}}\left[\mu (\nabla u+{\left(\nabla u\right)}^{T}-\frac{2}{3}\mu (\nabla \cdot u)I)\right]\right\}-\left(\kappa^{-1}\mu +\frac{Q_{br}}{\varepsilon_{c}^{2}}\right)u$$

where ρ is the density of the fluid (kg/m^3^), $\varepsilon_{c}$ is the column voidage (–), t is the time (s), $Q_{br}$ is the volumetric mass source of the fluid phase (kg/m^3^/s), κ denotes the permeability of the porous medium (m^2^), μ is the dynamic viscosity of the fluid (kg/(m·s)), p is the gauge pressure of the fluid flow (Pa), and u is the Darcy velocity (m/s).

The permeability of the packed bed of spherical particles can be described by the Blake–Kozeny equation (Rao et al., 2009) [22]:

(3) $$\kappa =\frac{d^{2}}{150}(\frac{\varepsilon_{c}}{1-\varepsilon_{c}}){}^{2}$$

where d is the average diameter of zeolite particles (m).

The mass source term $Q_{br}$ is defined as the mass deposit rate of nitrogen and oxygen inside the adsorbent to simulate the adsorption process (Rao, 2013) [28].

(4) $$Q_{br}=-\sum_{i=1}^{2}k_{i}M_{i}(c_{i}-c_{pi})$$

where $k_{i}$ is the macropore mass transfer rate of nitrogen and oxygen into zeolite particles (1/s), $M_{i}$ is the molecular weight of N_2_ and O_2_ (kg/mol), $c_{i}$ and $c_{pi}$ are the column gas concentration and particle concentration of nitrogen and oxygen, respectively (mol/m^3^) [29].

(5) $$k_{i}=\frac{60D_{i,j}^{e}}{K\cdot d^{2}}$$

where $D_{i,j}^{e}$ are the molecular diffusivity of N_2_ and O_2_ into the zeolite pore area (m^2^/s), K is the dimensionless Henry’s law constant for N_2_ and O_2_ (–) [30].

The molecular gas diffusivity was defined by the Chapman–Enskog Equation [31,32]. The parameters used in the Chapman–Enskog equation are shown in Table 4.

(6) $$D_{i,j}^{e}=\frac{5.953\times 10^{-24}}{p\sigma_{ij}^{2}\Omega_{D}}\sqrt{\frac{T^{3}}{M_{i}}+\frac{T^{3}}{M_{j}}}$$

where $\sigma_{ij}$ is the average collision diameter of components *i* and *j* (m), $\Omega_{D}$ is the collision integral (–), $M_{i}$ is the molecular mass of component *i* (kg/mol) and $M_{j}$ is the molecular mass of component *j* (kg/mol).

(7) $$\sigma_{ij}=\frac{\sigma_{i}+\sigma_{j}}{2}$$

where $\sigma_{i}$ is the collision diameter of component *i* (m) and $\sigma_{j}$ is the collision diameter of components *j* (m).

(8) $$\Omega_{D}=\frac{1.060}{T_{N}^{0.156}}+\frac{0.193}{e^{0.476T_{N}}}+\frac{1.036}{e^{1.530T_{N}}}+\frac{1.765}{e^{3.894T_{N}}}$$

where $T_{N}$ is the standardized temperature (–).

(9) $$T_{N}=\frac{T}{\sqrt{\frac{\varepsilon_{i}}{k_{b,i}}}\sqrt{\frac{\varepsilon_{i}}{k_{b,i}}}}$$

where $\varepsilon_{i}$ is the characteristic energy of component *i* (J), $\varepsilon_{j}$ is the characteristic energy of component *j* (J), $k_{b,i}$ is the Boltzman’s constant of component *i* (J/K) and $k_{b,j}$ is the Boltzman’s constant of component *j* (J/K).

The pressure drop is given by the Ergun equation [33]

(10) $$\frac{\partial p}{\partial z}=\frac{150\mu }{d_{p}^{2}}\frac{{\left(1-\varepsilon_{c}\right)}^{2}}{\varepsilon_{c}^{2}}u+\frac{1.75\rho }{d_{p}}\frac{\left(1-\varepsilon_{c}\right)}{\varepsilon^{3}}u|u|$$

where *z* represents the coordinate axis of the gas flow (m).

Input flow rate *F* is a function of pressure fitted with experimental data:

(11) F(p)=2.478−0.7251p

where *F* is the flow rate of inlet gas (L/min).

The gas distribution is solved in the Transport of Diluted Species in Porous Media Interface of COMSOL Multiphysics with convection and dispersion equations (COMSOL, 2016) [27].

(12) $$\frac{\partial c_{i}}{\partial t}-\nabla (D_{D}\cdot \nabla c_{i})+\nabla (uc_{i})=\frac{\left(1-\varepsilon_{c}\right)}{\varepsilon_{c}}(\varepsilon_{p}\frac{\partial c_{pi}}{\partial t}+\rho_{p}\frac{\partial q_{i}}{\partial t})$$

The total gas mass deposit rate from column void to zeolite particles are calculated from the combination of particle concentration dispersion rate and microparticle adsorption rate.

(13) $$\varepsilon_{p}\frac{\partial c_{pi}}{\partial t}+\rho_{p}\frac{\partial q_{i}}{\partial t}=k_{i}(c_{i}-c_{pi})$$

(14) $$\frac{\partial q_{i}}{\partial t}=k_{pi}(q_{i}^{*}-q_{i})$$

(15) $$q_{i}^{*}=q_{i}^{e}\frac{{\left(b_{i}P\omega_{i}\right)}^{1/n_{i}}}{1+\sum_{j=1}^{2}{\left(b_{j}P\omega_{j}\right)}^{1/n_{j}}}$$

(16) $$D_{D}=0.7D_{i,j}^{e}+0.5d_{p}u$$

where $k_{pi}$ is the micropore mass transfer rate of nitrogen and oxygen (1/s), $\rho_{p}$ is the density of zeolite particle (kg/m^3^), $q_{i}^{*}$ and $q_{i}$ are the equilibrium and actual adsorbed species to the zeolite, respectively (mol/kg), $\omega_{i}$ is the volume fraction of N_2_ and O_2_ (–), $q_{i}^{e}$ is the maximum surface excess of gas component onto zeolite pore area (mol/kg), $b_{i}$ is the adsorption constant of N_2_ and O_2_ (1/bar), $D_{D}$ is the axial dispersion coefficient (m^2^/s).

(17) $$k_{pi}=\frac{60D_{i}^{e}}{K\cdot d_{p}^{2}}$$

(18) $$D_{i}^{e}=\frac{\varepsilon_{p}}{\tau_{p}}\left(\frac{1}{\frac{1}{D_{k,i}^{e}}+\frac{1}{D_{i,j}^{e}}}\right)$$

where $D_{i}^{e}$ is the effective intra particle diffusivity of N_2_ and O_2_ into zeolite pore area (m^2^/s), $D_{k,i}^{e}$ is the effective Knudsen diffusivity of N_2_ and O_2_ into the zeolite pore area (m^2^/s) [34,35,36], $\tau_{p}$ is the pore tortuosity (–) [25].

The effective Knudsen diffusivity is given by [37]:

(19) $$D_{k,i}^{e}=\frac{d_{p}}{3}\sqrt{\frac{8RT}{\pi M_{i}}}$$

where $M_{i}$ is the molecular mass of nitrogen and oxygen (kg/mol), R is the ideal gas constant (J·K^−1^·mol^−1^), T is the column temperature (K).

*Initial condition of adsorption step*:

At *t* = 0, the initial pressure (gauge) = −0.82 (barg), the inflow is set at *p* = 1.79 (barg), $\omega_{N_{2}}$ = 0.79;

The initial adsorbed nitrogen concentration = 0.3172 (mol/kg), initial adsorbed oxygen concentration = 0.02564 (mol/kg);

*Initial condition of desorption step*:

At *t* = 0, the initial pressure = 1.79 (barg), the inflow is set at *p* (gauge) = −0.82 (barg), $\omega_{N_{2}}$ = 0.79;

The initial adsorbed nitrogen concentration = 1.333 (mol/kg), the initial adsorbed oxygen concentration = 0.1329 (mol/kg).

### 3.2. Geometry and Meshing of the Finite Element Algorithm

**Figure 8:**
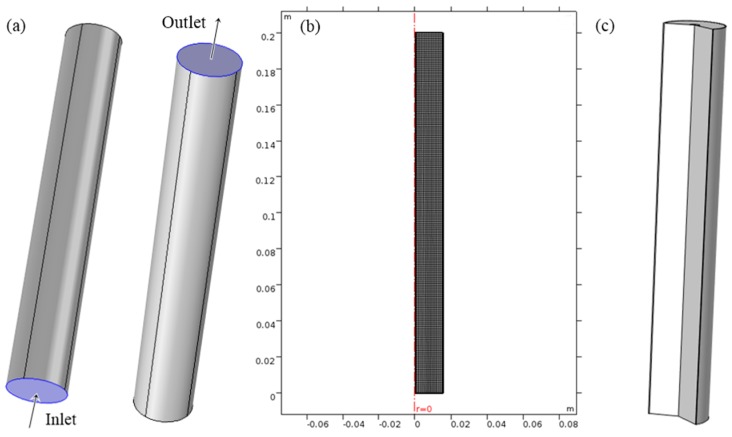
(**a**) Schematics of adsorption column; (**b**) refined mesh with axis of symmetry; (**c**) full model geometry.

The geometry of the column used for the model simulation was a cylinder with a height of 20 cm and a diameter of 3 cm. The gas source entered at the bottom of the column and enriched oxygen stream exited at the top of the column shown in Figure 8a.

The computational mesh was a swept rectangular mesh. The shape of the rectangular mesh fits both the shape of the axial gas dispersion pattern and the boundary layer condition. In order to find an appropriate mesh size, a mesh refinement study was undertaken with the difference function:

$$\%Difference=\frac{\left|\omega_{O_{2}}(mesh_{x})-\omega_{O_{2}}(mesh_{finest})\right|}{\omega_{O_{2}}(mesh_{finest})}\times 100\%,$$

where $\omega_{O_{2}}(mesh_{x})$ is the oxygen vol % of outflow at the mesh size *x* (mm) and $\omega_{O_{2}}(mesh_{finest})$ is the oxygen vol % of outflow at the finest mesh size (mm).

**Figure 9:**
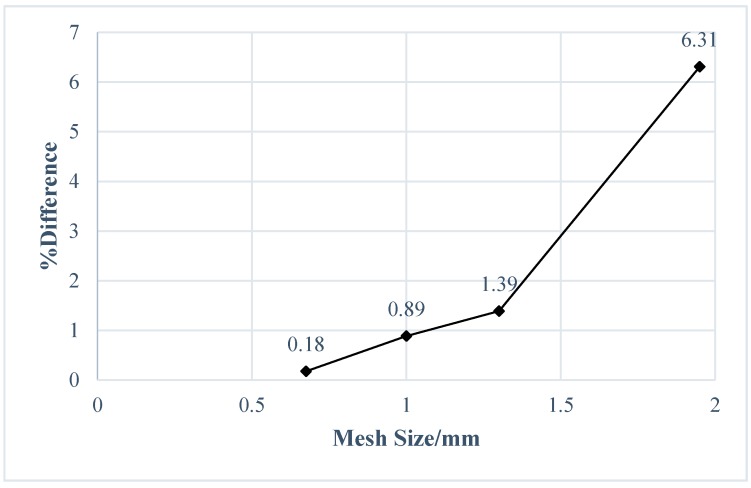
Mesh refinement study results.

Figure 9 shows the results of the mesh refinement study. The relative tolerance was set to 10^−6^ and largest %Difference of all the time points for each mesh size was evaluated. All the mesh sizes below 1 mm were fine enough using the criterion of less than 1% difference. The mesh size = 1 mm was selected and used for all the simulations. The refined mesh and geometry are shown in Figure 8b,c.

## 4. Results and Discussion

### 4.1. Concentrator Output

**Figure 10:**
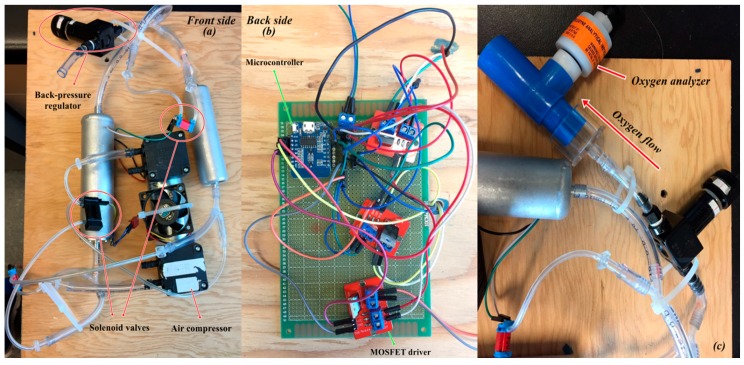
Photograph of oxygen concentrator prototype (**a**) front side, (**b**) back side, (**c**) Oxygen analyzer.

The photograph of the oxygen concentrator is shown in Figure 10 with the in-house made microcontroller circuit for the continuous production. The concentrator is tested with a vacuum/pressure swing cycle between −0.82 barg and 1.79 barg. The flowrate of output stream is 1.128 L/min and the average oxygen concentration peak is 91.64 vol %. The output oxygen concentration is almost stable at 1.79 barg, which means the desorption under a vacuum is effective and the desorption time is long enough to regenerate the zeolite. The enriched continuous oxygen stream can be conserved in a storage tank to produce a steady enriched air stream. The cyclic oxygen production test shows that the oxygen concentration decreased gradually to 80 vol % after 300 cycles. The adsorption column could be replaced every month for commercial portable oxygen concentrator development and the deactivated zeolite adsorbent could be regenerated in the oven at 450 °C with a continuous nitrogen flow to remove the desorbed contaminants.

### 4.2. Model Validation

**Figure 11:**
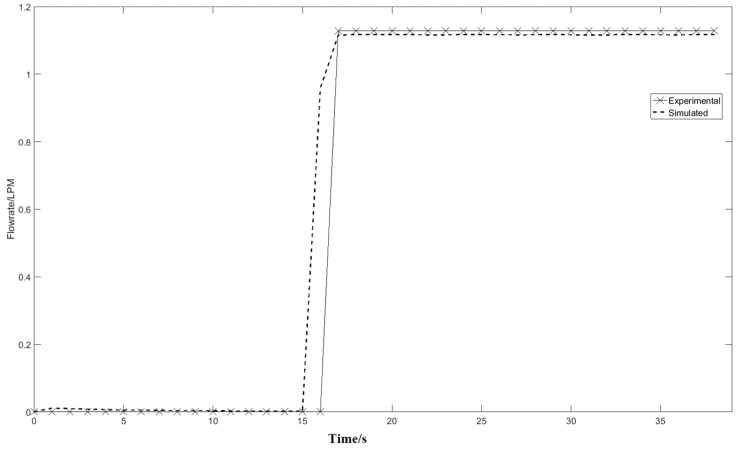
Comparison of the experimental and simulation results of the column outlet flow rate.

Figure 11 compares the experimental flow rate with the simulation results. The experimental and model output flowrates are 1.226 L/min and 1.24 L/min, respectively. The difference of the experimental data and simulation results is 1.1%, which means the modified Free and Porous Media Flow Interface is able to represent the outlet flow condition. Before 21 s, the adsorption column is in the pressurization stage without any gas flowing out through the outlet. The flow rate of the outlet is maintained at 1.226 L/min at the production stage, which means the same amounts of mass flow are entering and exiting the adsorption column to maintain the adsorption pressure after *t* = 21 s.

**Figure 12:**
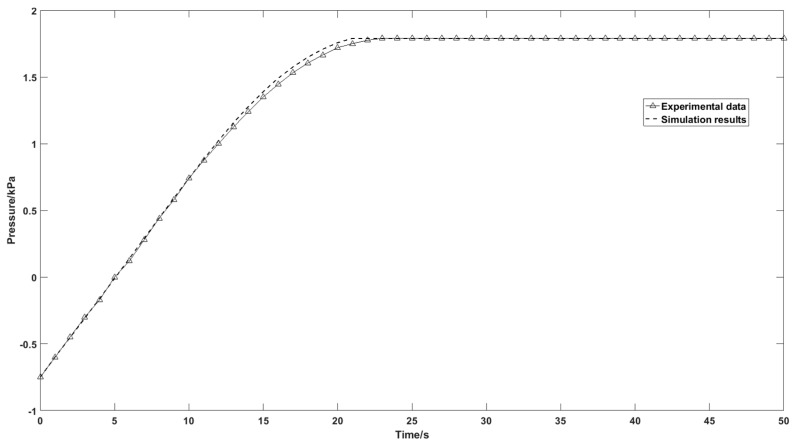
Comparison of the experimental and simulation results of the column outlet pressure.

Figure 12 shows the outlet pressure profile at different times during the adsorption step in the Free and Porous Media Flow Interface. The difference between the simulation results and the experimental data is negligible, which is significant for the adsorption function in the Transport of Diluted Species in Porous Media Interface. According to the outlet pressure and flowrate profile in Figure 11 and Figure 12, the model is able to represent the pressurization step flow condition of a portable oxygen concentrator with an air compressor instead of a constant flowrate simulation.

**Figure 13:**
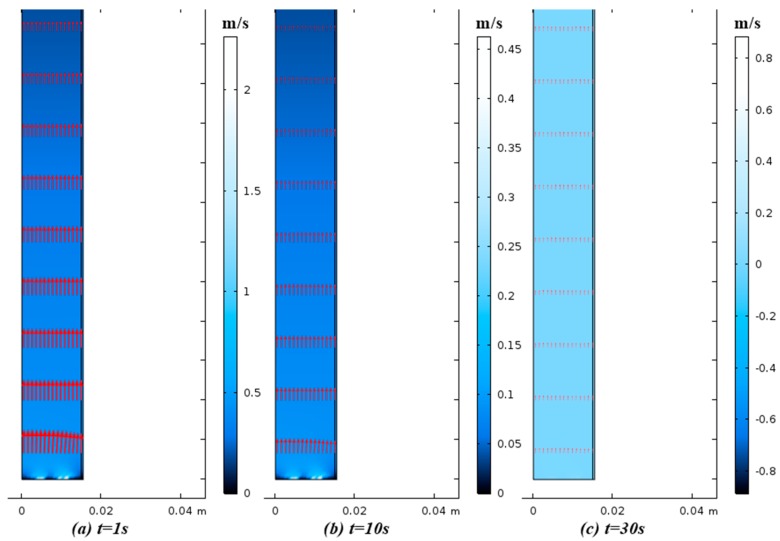
Internal velocity profile of adsorption column with inlet distributor at time = (**a**) 1 s; (**b**) 10 s; (**c**) 30 s.

Figure 13 shows the internal gas velocity profile at different times during the adsorption step. The ‘arrow’ surface represents the direction of velocity vector. When the bed is being pressurized, the velocity decreases from the inlet to the outlet due to the closed outlet boundary condition (Figure 13a,b). The velocity distribution at the production stage (Figure 13c) is uniform, which means the column is saturated and concentrated gas is transported from the outlet of the column at a constant pressure.

**Figure 14:**
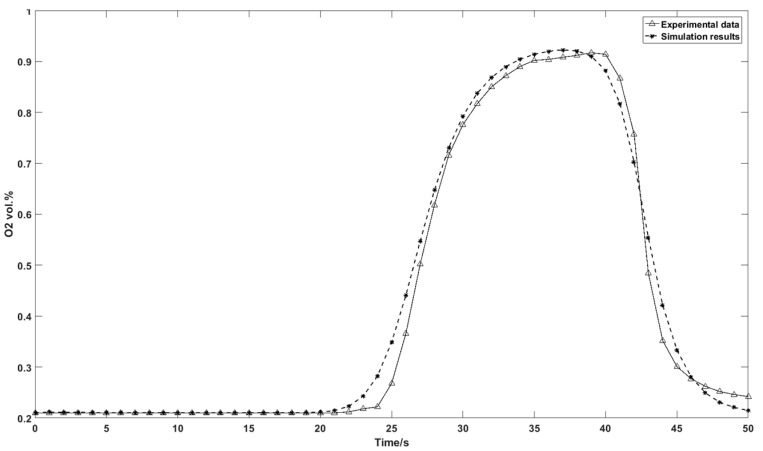
Experimental outflow oxygen concentration with simulation results.

Figure 14 shows the experimental and the simulation results of the outlet oxygen concentration at adsorption pressure = 1.79 barg and desorption pressure = −0.82 barg. The enriched oxygen product starts to flow out of the column at *t* = 21 s and the outlet oxygen concentration in the product flow reaches the peak at *t* = 35 s. The experimental data shows the same enriched tendency of oxygen in the outflow. Compared to the simulation result, a slight difference of the oxygen breakthrough curve is observed from the experimental oxygen sensor data. This could be explained by the inaccuracy of the multicomponent Sips adsorption equations due to the complexity of the heterogeneous zeolite porous surface.

**Figure 15:**
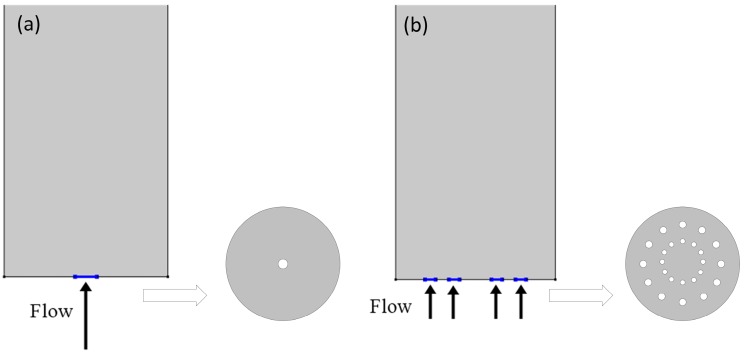
Schematics of inflow type at the inlet of the adsorption column: (**a**) central flow without inlet distributor; (**b**) distributed flow with inlet distributor.

**Figure 16:**
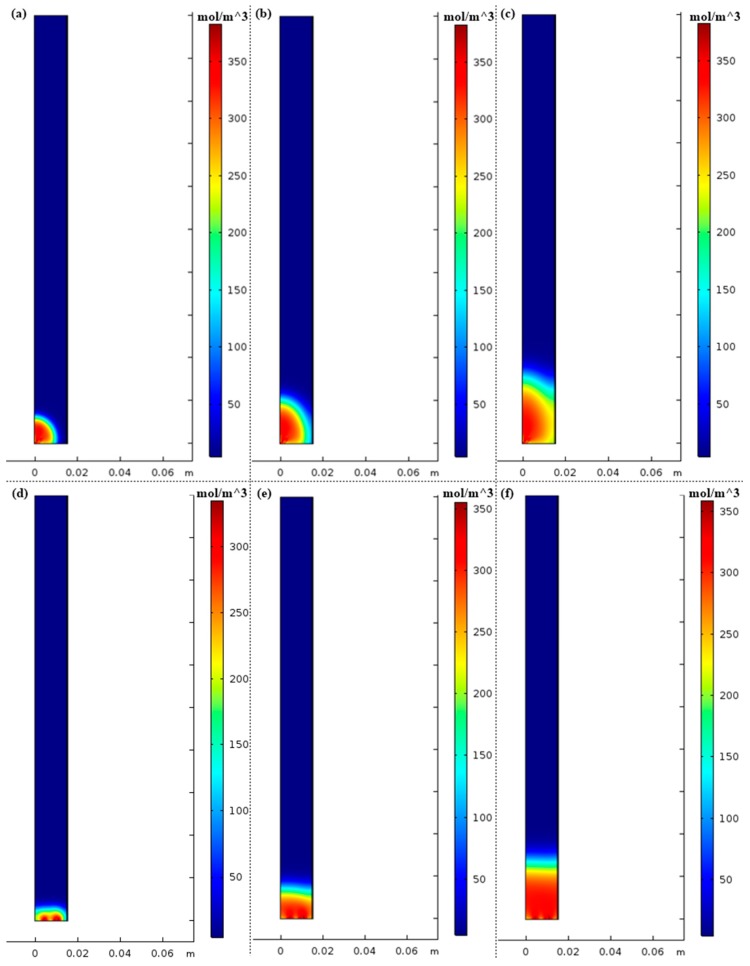
(**a**) N_2_ concentration profile without inlet distributor at *t* = 1 s; (**b**) N_2_ concentration profile without inlet distributor at *t* = 3 s; (**c**) N_2_ concentration profile without inlet distributor at *t* = 6 s; (**d**) N_2_ concentration profile with inlet distributor at *t* = 1 s; (**e**) N_2_ concentration profile with inlet distributor at *t* = 3 s; (**f**) N_2_ concentration profile with inlet distributor at *t* = 6 s.

**Figure 17:**
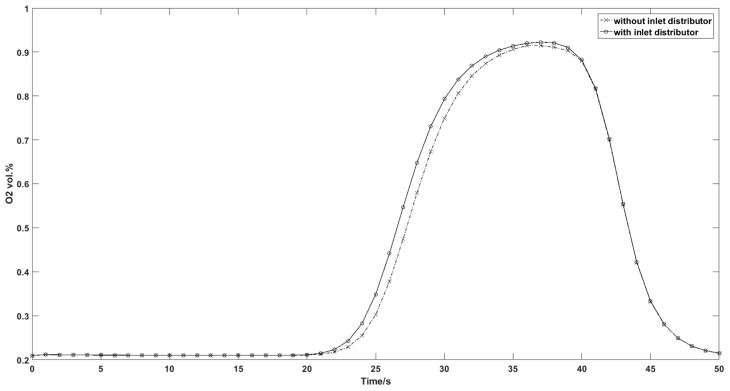
Simulation results of outflow oxygen concentration for different inflow type.

Two types of inlet flow distribution were investigated for the column adsorption test. Figure 15a illustrates the common central flow from tubing to the column inlet without inlet distributor and Figure 15b illustrates the distributed inflow with an inlet gas distributor. Figure 16 shows the N_2_ concentration profiles of two different inflow types at different times during the pressurization step. The distribution of the central flow is not uniform with a radial dispersion from column axis to the boundary while the distributed flow is closed to an ideal plug flow with negligible radial dispersion. Figure 17 compares the outflow concentration for distributed inflow and central flow. The results are in accordance with the experimental results in that the pressurization time of the column with central inflow is 1.6 s longer than that of the column with the distributed flow. The inlet gas distributor is significant for the inflow air and decreases the pressurization time for the adsorption cycle.

**Figure 18:**
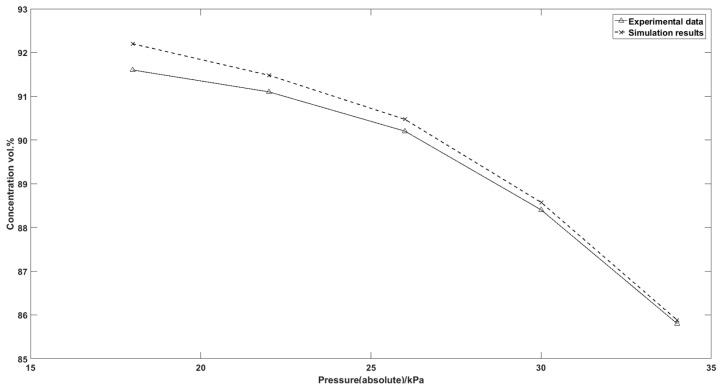
Output oxygen concentration peak from different vacuum swing range to 1.79 bar gauge pressure.

Figure 18 shows the concentration peak of the oxygen output breakthrough curve under different vacuum swing range. The nitrogen capacity of the zeolite decreases drastically when the pressure of vacuum port increases from 18 kPa (abs) to 34 kPa (abs). The deep vacuum desorption step is able to increase the productivity of the adsorption column. The simulation results also exhibit a good fitting precision of the experimental data with the maximum difference 0.67%. 

**Figure 19:**
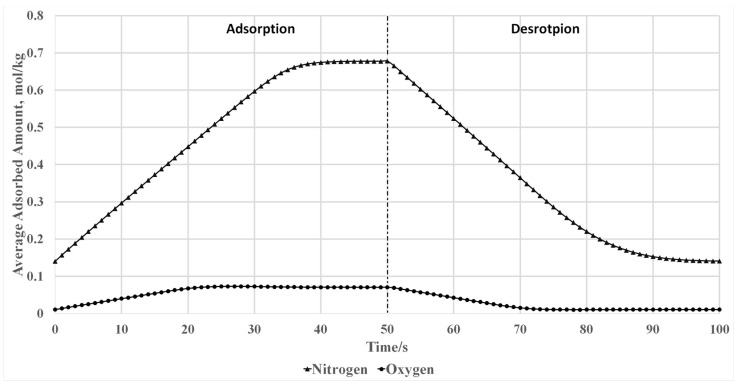
Simulation results of average adsorbed nitrogen and oxygen amount to the zeolite particles.

Figure 19 shows the average surface excess of the zeolite surface at the adsorption and desorption stage. The amount of N_2_ and O_2_ adsorbed to the zeolite particle gradually decreases when the adsorption column is connected to the suction port of the air compressor to create vacuum desorption conditions inside the column. N_2_ and O_2_ are released from the zeolite during the blowdown and purge stages, which generates a clean bed for the next adsorption cycle.

## 5. Conclusions

A portable two-column pressure vacuum swing adsorption (PVSA) oxygen concentrator was built in our laboratory and its performance was modeled by solving the continuity equations and the momentum equation in COMSOL Multiphysics. The model was able to simulate the microporous adsorption of nitrogen under a dry and CO_2_ free compressor flow. The velocity and oxygen concentration profiles were investigated at every time step (Δt = 0.1 s) during the four operation stages: pressurization, production, blowdown, and desorption. With the process optimization, it was possible to get better operational parameters and design characteristics to improve the performance of the concentrator units and meet the terminal requirements of oxygen supply (O_2_ concentration ~88–92 vol %). The valves and the compressor of the prototype are controlled by a microcontroller program for continuous oxygen production. The concentrator is a lightweight, miniature unit suitable for outside oxygen supplement applications.

## Figures and Tables

**Table 1:** The valve operation sequence for the PVSA cycle.

Step	Stage	Column 1		Stage	Column 2	
		Valve 1	Valve 3		Valve 2	Valve 4
1	Pressurization	P1	P1	Blowdown	P2	P2
2	Production	P1	P1	Vacuum	P2	P1
3	Blowdown	P2	P2	Pressurization	P1	P1
4	Vacuum	P2	P1	Production	P1	P1

P1, P2 represents valve position 1 and 2.

**Table 2:** BET adsorption results of JLOX-101 and 13X-BJ-DF.

Zeolite	JLOX-101	13X-BJ-DF
BET surface area (m^2^/g)	574.77	522.93
Micropore area (m^2^/g)	515.39	477.04
Single Point Adsorption Total Pore Volume (cm^3^/g)	0.31	0.29
Micropore Volume (cm^3^/g)	0.24	0.22
Adsorption Average Pore Diameter (Å)	21.26	22.36

**Table 3:** Characteristics of adsorbent, desiccant and adsorption column [17,22,23,24,25].

Adsorbent	Zeolite LiX	Unit
Average zeolite particle size	600	µm
Average microparticle size	5.75	µm
Zeolite bulk density	790	kg/m^3^
Particle voidage, $\varepsilon_{p}$	0.35	1
Maximum surface excess of N_2_, $q_{N_{2}}^{e}$	3.42	mol/kg
Maximum surface excess of O_2_, $q_{O_{2}}^{e}$	6.06	mol/kg
Adsorption constant, $b_{N_{2}}$	0.09	1/bar
Adsorption constant, $b_{O_{2}}$	0.02	1/bar
Pore tortuosity, $\tau_{P}$	3	-
BET Surface Area	574.77	m^2^/g
Micropore Volume	0.240007	cm^3^/g
Adsorption Average Pore Diameter	21.2609	Å
**Desiccant Type**	**Activated Alumina**	
Particle density	765	kg/m^3^
Average desiccant particle size	600	µm
Adsorption Column	-	-
Length	10	cm
Inside radius	1.5	cm
Column voidage, $\varepsilon_{c}$	0.36	1
Material	aluminum	-

**Table 4:** Characteristics parameters of nitrogen and oxygen [38,39,40].

Adsorbent	Zeolite LiX	Unit	Reference
Dynamic viscosity of the fluid, μ	1.84 × 10^−5^	Pa·s	Smits et al., 2006
Dimensionless Henry’s law constant of N_2_, $K_{N_{2}}$	1.5 × 10^−2^	-	Sander, 2015
Dimensionless Henry’s law constant of O_2_, $K_{O_{2}}$	3.2 × 10^−2^	-	
Average collision diameter of N_2_, $\sigma_{N_{2}}$	3.67	10^−10^ m	Bird et al., 2007
Average collision diameter of O_2_, $\sigma_{O_{2}}$	6.06	10^−10^ m	
Characteristic energy of N_2_, $\varepsilon_{N_{2}}/k_{b,N_{2}}$	99.8	K	
Characteristic energy of O_2_, $\varepsilon_{N_{2}}/k_{b,N_{2}}$	113	K	
Molecular Weight, M_N2_	28.01	10^−3^ kg/mol	
Molecular Weight, M_O2_	31.99	10^−3^ kg/mol	
Gas constant, R	8.31	J/(K·mol)	
